# ChewNet: A multimodal dataset for invivo and invitro beef and plant-based burger patty boluses with images, texture, and force profiles

**DOI:** 10.1016/j.dib.2025.111890

**Published:** 2025-07-16

**Authors:** Isurie Akarawita, Bangxiang Chen, Jaspreet Singh Dhupia, Martin Stommel, Weiliang Xu

**Affiliations:** a Department of Mechanical and Mechatronics Engineering, The University of Auckland, Auckland 1010, New Zealand; b Department of Mechatronics and Robotics, School of Advanced Technology, Xi’an Jiaotong-Liverpool University, Suzhou 215123, China; c Department of Electrical and Electronic Engineering, Auckland University of Technology, Auckland 1010, New Zealand

**Keywords:** Chewed food bolus, Images, Textural analysis, Mastication, Robotic chewing

## Abstract

This dataset presents a comprehensive multimodal collection of data acquired from the chewing of beef and plant-based burger patties using both human participants (Invivo) and a biomimicking robotic chewing device (Invitro). The primary objective of the data collection was to discover relationships regarding the change in food bolus properties with the number of robotic chewing cycles as the human swallowing threshold is achieved, which will facilitate the development of deep learning models capable of predicting mechanical and textural properties of chewed food boluses from images. In the in-vivo experiments, expectorated bolus samples were collected from three healthy adult male participants, who chewed food samples until just before swallowing. The chewed boluses were then imaged using a 12MP camera and a flatbed scanner, followed by Texture Profile Analysis (TPA) to measure texture parameters. The dataset comprises two main folders Invivo and Invitro. The Invivo data thus comprises high-resolution images and corresponding TPA metrics at the near-swallowing stage. In the Invitro experiments, a 3 degree of freedom linkage chewing robot (ChewBot) with a soft robotic oral cavity was used to simulate human mastication. The robot performed controlled mastication using different molar trajectories that varied in lateral shearing effect. Food samples were chewed for up to 40 chewing cycles, with artificial saliva introduced at 10 % of the food's weight. For each experimental condition, the dataset includes real-time images captured immediately after each the robotic chewing cycle, force profiles recorded at 100 ms intervals during the chewing, and TPA metrics of the resulting bolus after every 5 chewing cycles. This dataset has significant reuse potential in various fields. In food science, it can support studies on the mechanical breakdown of meat and meat alternatives, aiding in the reformulation of plant-based foods to better mimic desirable animal-based food textures. This dataset supports rehabilitation in health sciences by aiding personalized diet design for individuals with jaw disorders or dysphagia and guiding texture-appropriate menu options for patients. In robotics and artificial mastication, it informs the development of chewing systems. It also enables machine learning applications for predicting food texture from images, allowing automated, non-invasive analysis.

Specifications Table

**Table:** 

Subject	Engineering & Materials science
Specific subject area	Robotic and human mastication analysis using multimodal data for texture profiling and machine learning in food and sensory sciences.
Type of data	Food bolus images in .png, TPA data as tables in excel and pdf files, ChewBot’s chewing force data as tables in excel files.
Data collection	This study examined the Invivo and Invitro mastication characteristics of two commercially available products: the Impossible Plant-Based Burger Patties [1] and Silver Fern Farms Pure 97 % Beef Sliders with Brisket [2]. The patties were oven-cooked to 74 °C internal temperature, cooled to room temperature, and portioned into consistent 2 g samples. For the Invivo experiments, three participants first chewed and swallowed three samples of each food type using one side of their mouth while being video recorded to count chewing cycles. They then repeated the process with additional samples, expectorating the boluses just before swallowing. The collected boluses underwent comprehensive analysis: each was weighed using precision scales, imaged at a constant angle and distance using a 12MP camera and a flatbed scanner, and subjected to TPA to quantify key textural parameters at the swallowing point. For the Invitro chewing, this study used a 3-degree-of-freedom biomimicking chewing robot (ChewBot) with a soft robotic oral cavity to simulate human mastication. The robot’s oral cavity, powered by pneumatic bellows, reposition food between occlusions, while an automated syringe injects saliva at a controlled rate (0.2 mL per sample, 10 % of food weight). Invitro experiments tested two trajectories: T1 (more vertical trajectory with minimal shear) and T13 (more lateral trajectory with maximal shear) using 3-second chewing cycles up to 40 cycles (5, 10, 15, …, 40), with increments of 5. Each experiment setting was repeated 5 times. Each food sample was individually chewed for the specified cycles by the robot, and the resulting boluses were analysed.
	Post-chew data include: (1) high-resolution images of each bolus captured immediately after each jaw opening; (2) force profiles (in Newtons) recorded at 100 ms intervals during mastication; and (3) TPA metrics after every 5 chewing cycles until 40 chewing cycles.
Data source location	Data was collected from Department of Mechanical and Mechatronics Engineering, The University of Auckland, Auckland 1010, New Zealand. Latitude: −36.8529542 and Longitude: 174.7698901 Data are stored in Mendeley Data Repository.
Data accessibility	Repository name: Mendeley Data Data identification number: DOI: 10.17632/kk9b7g3nv9.2 Direct URL to data: https://data.mendeley.com/datasets/kk9b7g3nv9/2
Related research article	None

## 1 Value of the Data

**Fig. 1:**
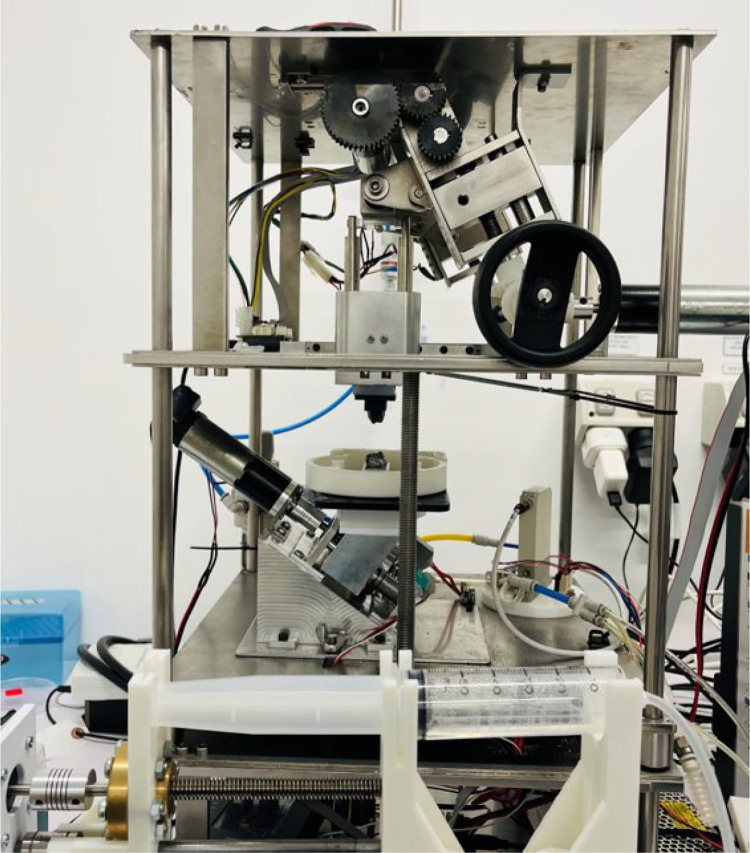
ChewBot: the chewing robot.
• Comprehensive mastication analysis for food innovation: This dataset provides a comprehensive set of TPA parameters (hardness, adhesiveness, cohesiveness, springiness, etc.) and high-resolution visual images of food boluses at sequential mastication points. By analyzing parameters such as chewing cycle counts, bolus formation patterns, and Invivo swallowing thresholds, researchers can identify key differences between natural and artificial mastication. Direct comparison of beef and plant-based samples under identical chewing conditions provides critical insights for replicating meat-like textures in alternatives. By analyzing parallel breakdown patterns from initial chewing as shown in Fig. 2, researchers can pinpoint exact textural gaps in plant-based formulations and develop solutions to match real meat's mouthfeel and structural integrity. Furthermore, food manufacturers and scientists can use these metrics to develop new formulations, particularly for specialized diets such as softer textures for elderly populations or more resilient structures for better chewing satisfaction.

• Cutting-edge biomimetic chewing robots’ food bolus formulation dataset: This study presents the Invitro data from one of the few biomimicking chewing robots available worldwide, with its design and applications previously documented in peer-reviewed journals [3]. Fig. 1 illustrates the ChewBot. This invitro dataset under the ChewNet, is unique as no other datasets currently exist that capture data from a chewing robot [4]. It provides unique insights into food bolus properties at various chewing stages, offering researchers in robotics and food science a valuable reference for developing and refining artificial mastication systems. By comparing the robot's performance with human chewing data, scientists can better understand how to simulate natural oral processing, which is crucial for advancing food texture analysis, dental research, and nutritional studies. The detailed bolus characteristics (particle size distribution, moisture content, robots’ force profiles, and chewed bolus TPA data) serve as benchmarks for improving robotic chewing mechanisms and validating new simulator designs.

**Fig. 2:**
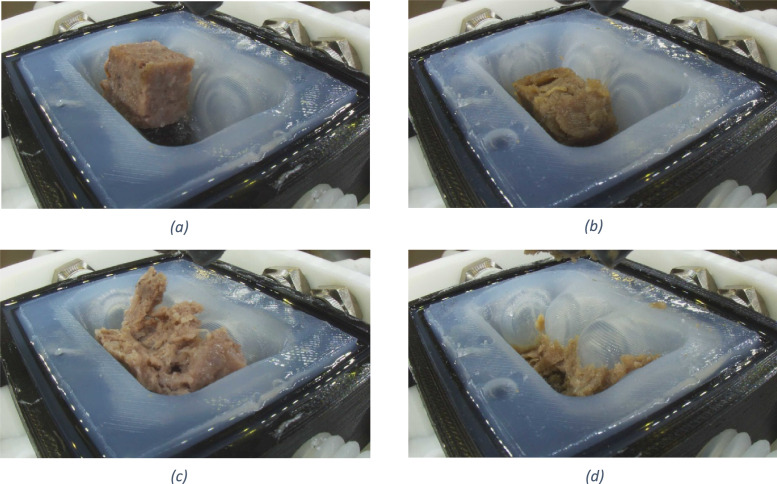
Comparing beef and plant-based burger Pattie samples (a) Beef sample before chewing (b) Plant sample before chewing (c) Beef sample after 24 chewing cycles and (d) Plant sample after 24 chewing cycles.
• Pioneering image-based texture analysis: This dataset can be used for novel research in non-destructive food texture evaluation through computer vision approaches. While recent studies have demonstrated moisture content evaluation from food images [5], this comprehensive collection of high-resolution bolus images paired with precise TPA measurements and ChewBot’s force profiles, can create the first benchmark for correlating visual features with fundamental texture parameters. The precisely timed image capture at critical mastication stages (1–40 chews) provide a unique opportunity to develop machine learning models that can estimate texture properties purely from visual data. Building upon recent advances in Grey Level Co-occurrence Matrix (GLCM) based textural analysis of in vitro chewed boluses, which revealed consistent trends in GLCM energy, homogeneity, and dissimilarity across chewing cycles [6], this Invitro dataset of ChewNet offers a more extensive visual record aligned with physical measurements, enabling deeper modeling of the dynamic relationship between mastication stages and visual texture features. Such image-based analysis methods could revolutionize food quality control by enabling rapid, non-invasive texture assessment in production lines. This dataset fills a critical gap in computer vision for food science by providing ground truth TPA data for algorithm training and validation.
• Foundational resources for AI driven food science and methodological transparency: The annotated dataset (chewing cycles, TPA results, and bolus images) serves as a training ground for machine learning algorithms in food science applications. As AI models become increasingly integrated into healthcare and robotic systems, they are also becoming vulnerable to emerging security threats such as data poisoning, adversarial examples, and model inversion attacks. Ensuring the integrity and authenticity of training data, such as the ChewNet dataset, is therefore critical. A previous study presented a deep learning system using Mask R-CNN to quantify and achieve real-time evaluation of the chewing process for peanut samples. Such study is an example of how computer vision and machine learning can be applied to analyze food properties [7]. Similarly, researchers can use this dataset to develop predictive models for food breakdown behavior, automate texture classification systems, or simulate mastication outcomes without extensive human trials. The detailed documentation of experimental protocols including human participation ethics approval, food sample preparations, chewing experiments, and robotic simulation parameters provides a transparent framework for reproducible research in mastication dynamics. This methodological clarity supports interdisciplinary collaboration between food scientists, engineers, and nutritionists. The dataset's structure facilitates easy integration with computational modeling approaches in food research.

## 2 Background

Chewing robots represent a growing research area aimed at developing biomimetic oral cavities. Several robots such as the Tri-chewer [8], iBOMS-III [9], and 6-axis bionic chewing robot [10] have been engineered to match human mastication. In particular, the three-linked chewing robot known as ChewBot, was designed to reproduce molar trajectories, salivary action, force profiles and bolus repositioning within the oral cavity [3]. With these mechanical systems now available, current efforts focus on replicating human-like bolus formation. Capturing high-resolution images of in vitro chewed boluses at each cycle is critical for characterizing chewing behaviour and progression. However, the current lack of publicly available datasets hinders research progress in this area. To address this gap, we present the Invitro dataset under the ChewNet dataset [4] a first-of-its-kind multimodal dataset. The Invitro dataset includes two food substrates plant-based and beef burger patties processed through sequential chewing cycles ranging from 1 to 40. For each cycle, we collected bolus images and force profiles, and at five-cycle intervals we recorded textural measurements. Along with Invitro dataset, ChewNet contains an Invivo experimental dataset which includes images of food boluses at the swallowing stage and it’s corresponding TPA measurements. The purpose of this whole multimodal dataset is to enable development of deep learning models to evaluate chewing dynamics and forecast subsequent chewing patterns, offering a synchronized visual, mechanical and textural record of bolus formation in a human analogous oral environment.

## 3 Data Description

**Fig. 3:**
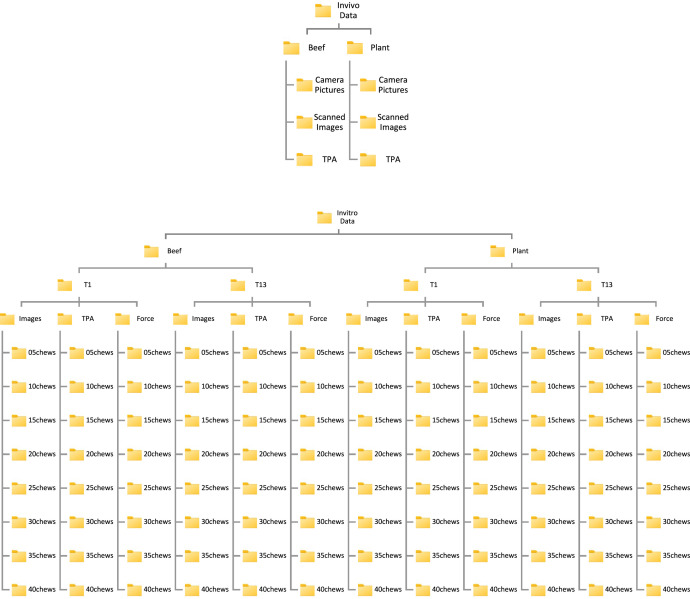
File structures of invivo and invitro dataset.

Below is an in-detail description of the data folders, subfolders, and individual files available in the Mendeley Data repository. The repository contains 1 main folder, named as ChewNet. In it contains the two main folders named as Invivo Data and Invitro Data. The file structure of both these folders are illustrated in Fig. 3.

**INVIVO DATA**

### 3.1 Image folders

**Fig. 4:**
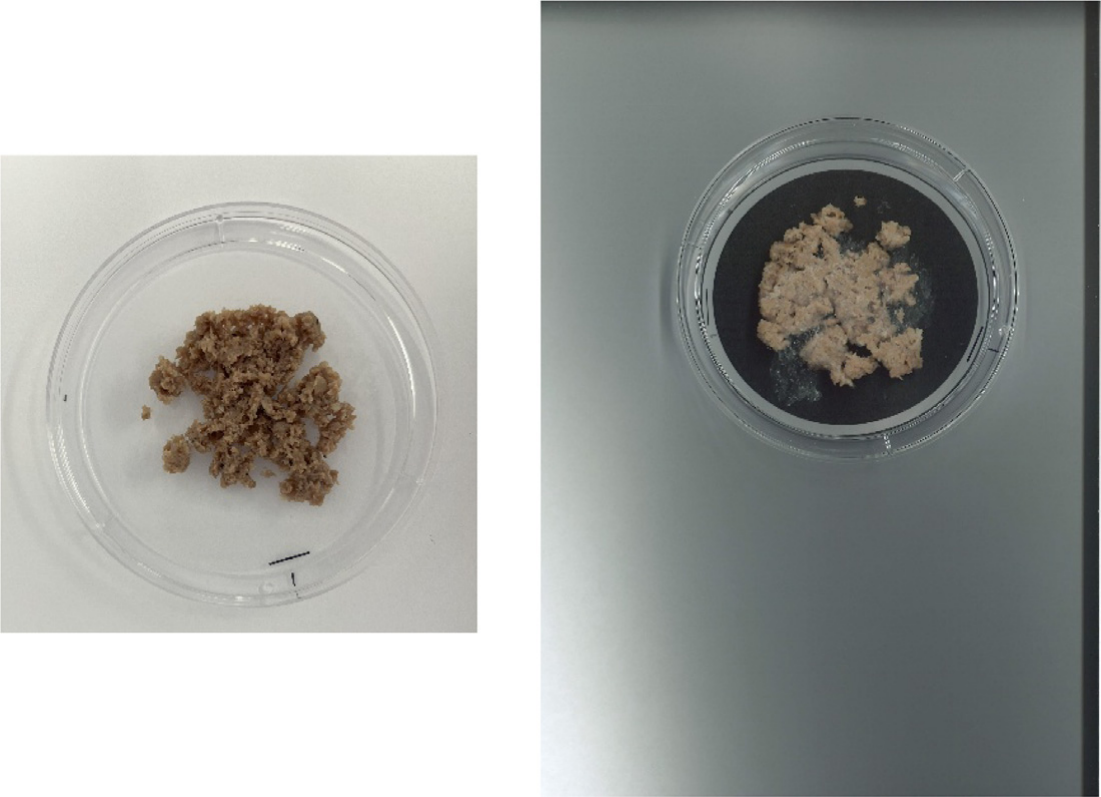
BIS, A beef bolus captured from phone (left image) and captured from the scanner (right image).

The images document food boluses expectorated by human participants immediately before swallowing, captured in petri dishes marked with a 1 cm scale for spatial calibration during image processing (Table 1). This dataset contains dual imaging systems to visualize the food bolus: (1) an iPhone 13 mini captured high-resolution images (2800×2800 pixels, .JPG format) under controlled lighting conditions, and (2) a Ricoh color scanner produced higher-detail scans (3496×4964 pixels, .JPEG format) with consistent flatbed positioning. A sample of the two image types are shown in Fig. 4.

**Table 1:** Contents of the invivo data folder.

Invivo data folder					
Beef			Plant		
Pictures	Scanner Images	TPA	Pictures	Scanner Images	TPA
9 Images	9 Images	9 excel and 9 pdf files	9 Images	9 Images	9 excel and 9 pdf files

### 3.2 TPA folders

The textural properties of food boluses produced by the ChewBot were measured using the Texture Profile Analysis using a Brookfield TexturePro CT V1.10 system. To maintain the consistency, TPA was conducted on a block-shaped food samples of height 10 mm [11]. The test involved two compression cycles to 80 % deformation with a trigger load of 7 g and a test speed of 1 mm/s [12]. A TA3/100 probe and TA-RT-KIT fixture were used with a 10,000 g load cell. Data were recorded at 10 points/*sec*. All these specifications are given along with each file available in the TPA folder as given in Table 1.

The TPA folder includes paired .xls (Excel) and .pdf files containing identical data, provided in dual formats for user convenience. The files adhere to the same standardized naming convention as the corresponding bolus images, ensuring seamless cross-referencing. Within these files, users will find:

• Sample details: Identification of the chewed food specimen
• Testing protocol: Methodology for TPA
• Textural parameters: Quantitative results for all TPA metrics

### 3.3 Naming convention

Each file follows a systematic naming convention. Images are named inside the image folder as, e.g., BIS7, where *B* = Beef or *P* = Plant, *I* = Invivo, *S* = Swallowing stage. The numbering system encodes participant specific data. There were 3 participants and each performed 3 repetitions for each food type. 1–3 represent the first participant, 4–6 the second, and 7–9 the third. Thus, in the chosen example, the name corresponds to the image of bolus collected from the third participant. The TPA folder maintains the same naming convention for both PDF and Excel files corresponding to each image, thereby facilitating reliable traceability across all replicates.

**INVITRO DATA**

### 3.4 Image folders

**Fig. 5:**
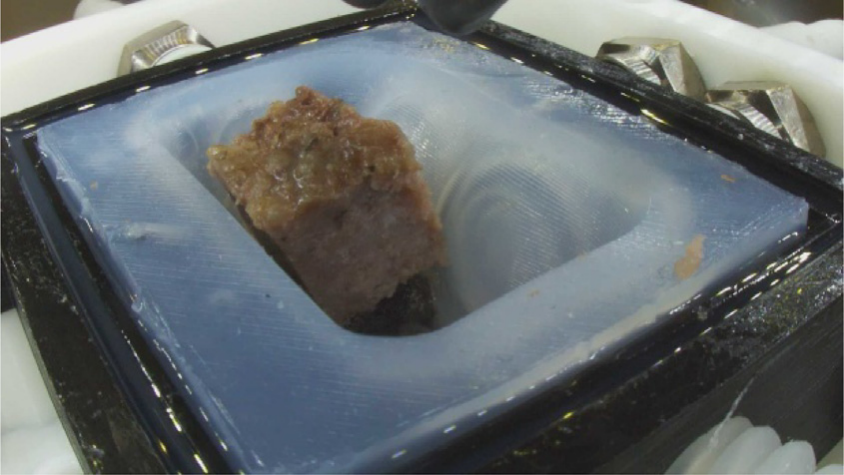
Image corresponding to BT1N0#5C15.

**Fig. 6:**
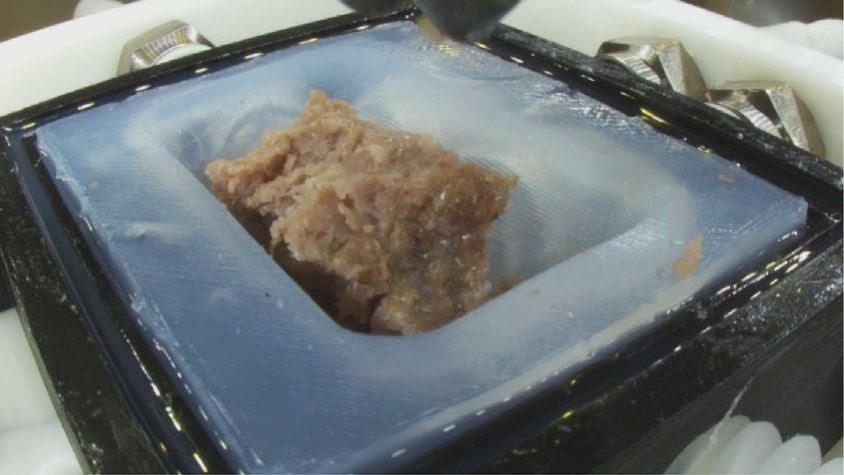
Image corresponding to BT1N10#5C15.

The image files show the food bolus immediately after the robotic jaw opens, revealing its post-occlusal state. During the occlusal phase (when molars collide), the bolus remains concealed beneath the upper molar. These images are critical for analyzing the structural break down for the chewed food bolus formation at each chewing cycle. Each image folder shown in Table 2 and Table 3, contains .PNG files of resolution 1920×1080 pixels. Two sample images are shown here in Fig. 5, Fig. 6.

**Table 2:** Contents of the invitro data → beef folder.

	Invitro data folder					
	Beef					
	T1			T13		
	Images	TPA	Force	Images	TPA	Force
After 5 Chewing Cycles	30 Images	5 excel and 5 pdf files	5 excel files	30 Images	5 excel and 5 pdf files	5 excel files
After 10 Chewing Cycles	55 Images	5 excel and 5 pdf files	5 excel files	55 Images	5 excel and 5 pdf files	5 excel files
After 15 Chewing Cycles	80 Images	5 excel and 5 pdf files	5 excel files	80 Images	5 excel and 5 pdf files	5 excel files
After 20 Chewing Cycles	105 Images	5 excel and 5 pdf files	5 excel files	105 Images	5 excel and 5 pdf files	5 excel files
After 25 Chewing Cycles	130 Images	5 excel and 5 pdf files	5 excel files	130 Images	5 excel and 5 pdf files	5 excel files
After 30 Chewing Cycles	155 Images	5 excel and 5 pdf files	5 excel files	155 Images	5 excel and 5 pdf files	5 excel files
After 35 Chewing Cycles	180 Images	5 excel and 5 pdf files	5 excel files	180 Images	5 excel and 5 pdf files	5 excel files
After 40 Chewing Cycles	205 Images	5 excel and 5 pdf files	5 excel files	205 Images	5 excel and 5 pdf files	5 excel files

**Table 3:** Contents of the invivo data → plant folder.

	Invitro data folder					
	Plant					
	T1			T13		
	Images	TPA	Force	Images	TPA	Force
After 5 Chewing Cycles	30 Images	5 excel and 5 pdf files	5 excel files	30 Images	5 excel and 5 pdf files	5 excel files
After 10 Chewing Cycles	55 Images	5 excel and 5 pdf files	5 excel files	55 Images	5 excel and 5 pdf files	5 excel files
After 15 Chewing Cycles	80 Images	5 excel and 5 pdf files	5 excel files	80 Images	5 excel and 5 pdf files	5 excel files
After 20 Chewing Cycles	105 Images	5 excel and 5 pdf files	5 excel files	105 Images	5 excel and 5 pdf files	5 excel files
After 25 Chewing Cycles	130 Images	5 excel and 5 pdf files	5 excel files	130 Images	5 excel and 5 pdf files	5 excel files
After 30 Chewing Cycles	155 Images	5 excel and 5 pdf files	5 excel files	155 Images	5 excel and 5 pdf files	5 excel files
After 35 Chewing Cycles	180 Images	5 excel and 5 pdf files	5 excel files	180 Images	5 excel and 5 pdf files	5 excel files
After 40 Chewing Cycles	205 Images	5 excel and 5 pdf files	5 excel files	205 Images	5 excel and 5 pdf files	5 excel files

### 3.5 TPA folders

Each TPA folder as shown in Table 2 and Table 3 includes paired .xls (Excel) and .pdf files containing identical data, provided in dual formats for user convenience. The files adhere to the same standardized naming convention as the corresponding bolus images, ensuring seamless cross-referencing. This structured approach facilitates efficient data retrieval and analysis across different software platforms.

### 3.6 Force folders

**Fig. 7:**
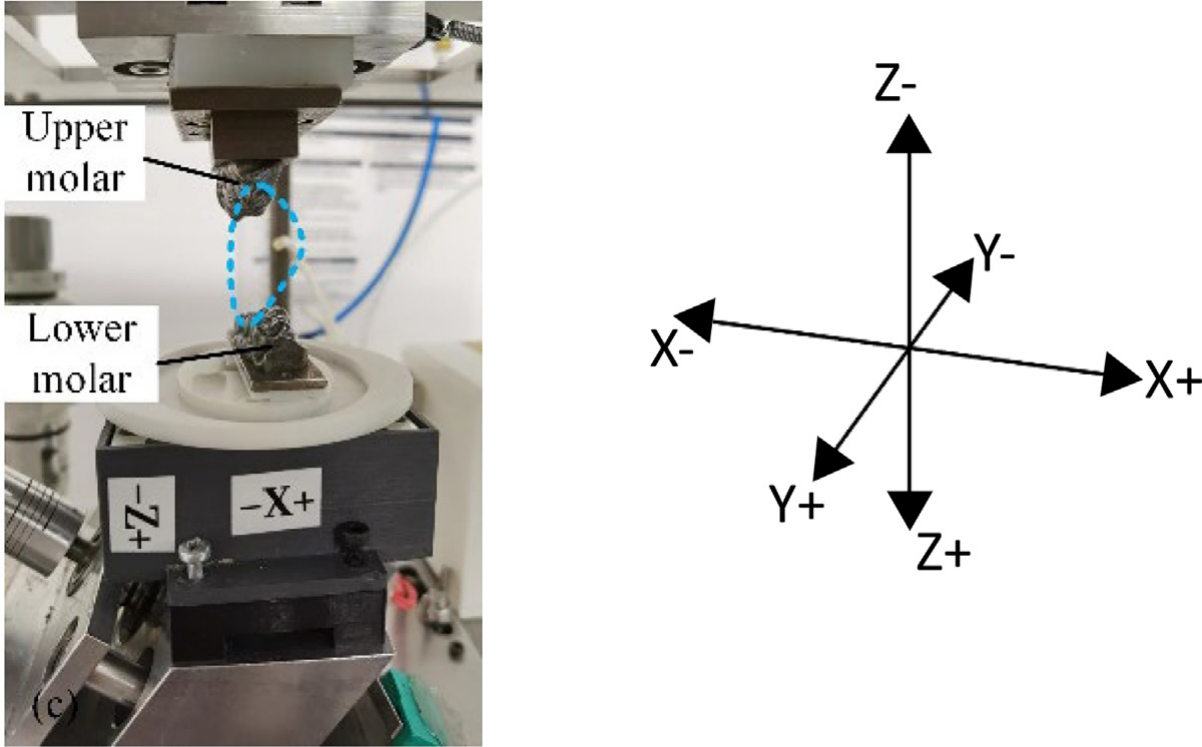
Chewing force directions.

The force data folders contain .xlsx files documenting the chewing robot's force exertion during mastication, with measurements recorded at 100 ms intervals. The files are organized into five excel sheet columns: Column A specifies the timestamp, while Columns B, C, and D record the force components (in Newtons) along the X, Y, and Z axes respectively, with Column E calculating the resultant total force magnitude. This force data reflects the mechanical interaction between the food sample and the molars during each chewing cycle. A load cell is positioned beneath the lower molar to measure forces, with the X and Z directions indicated in Fig. 7. The positive Y-direction is defined as extending outward from the plane of the image.

### 3.7 Naming convention

The Image, TPA, and Force folders contain subfolders as shown in Fig. 3. Consider the example of images collected during chewing of Beef (B) with vertical trajectory (T1) under the folder Images. The 05 chews subfolder refers to the images collected for 5 chewing cycles. The image name BT1NX#YC5, where the NX = N0 to N5 refers to the image collected after X chewing cycles and #*Y* = #1 to #5 refers the experiment repetitions. Thus, the overall images collected in this folder are 30. For folders with higher number of chews, NX will start from 0 chews to the maximum number of chews as listed in the folder name.

The naming convention of the images in the folder is as follows. For example, BT1N2#3C10: *B* = Beef or *P* = Plant-based, T1 = Robot Chewing Trajectory is 1 which is the vertical trajectory with more biting and compressive or T13 = Robot Chewing Trajectory is 13 which is a lateral trajectory with more grinding and shearing, N2 = Current chewing cycle is 2, #3 = 3rd sample out of the 5 repetitions, C10 = Final number of chewing cycles is 10 for this experiment setting. It is this same naming convention used all throughout the TPA and Force folders to ensure seamless cross referencing.

## 4 Experimental Design, Materials and Methods

### 4.1 Food sample preparation

**Fig. 8:**
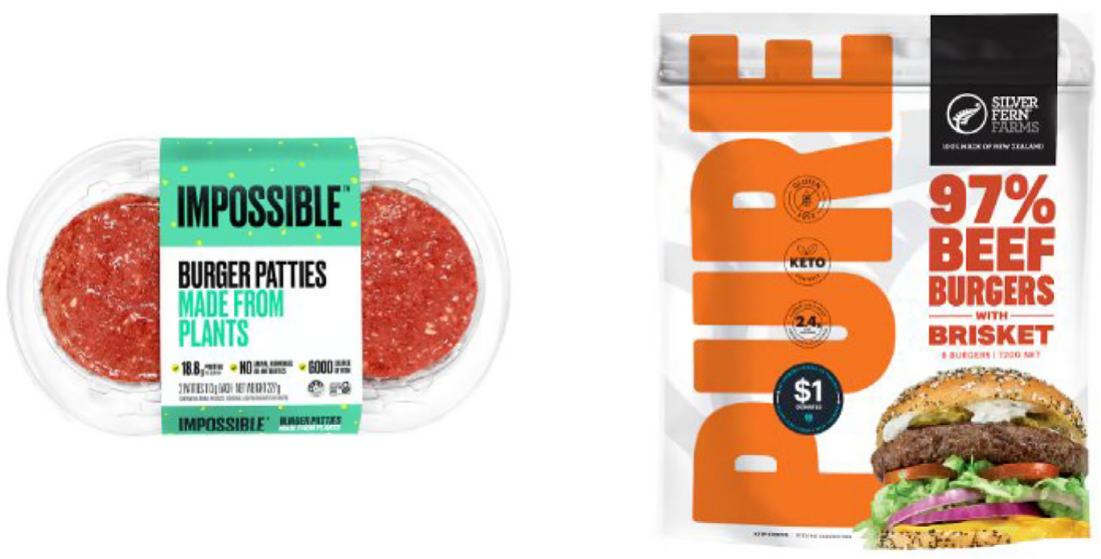
Plant-based and beef burger Patties.

Two commercially available products tested in this research are the Impossible Plant-Based Burger Patties [1] and Silver Fern Farms Pure 97 % Beef Sliders with Brisket [2] which are shown in Fig. 8. These burger patties were cooked in the oven at 200 °C until the temperature in the centre reached 74 °C. The cooked patties were cooled down to room temperature. Prior to the test, the patties were cut into small pieces or also known as samples, of roughly 1 cm × 1 cm × 1.5 cm and weighing around 2 g each for consistency.

### 4.2 Human participants

**Fig. 9:**
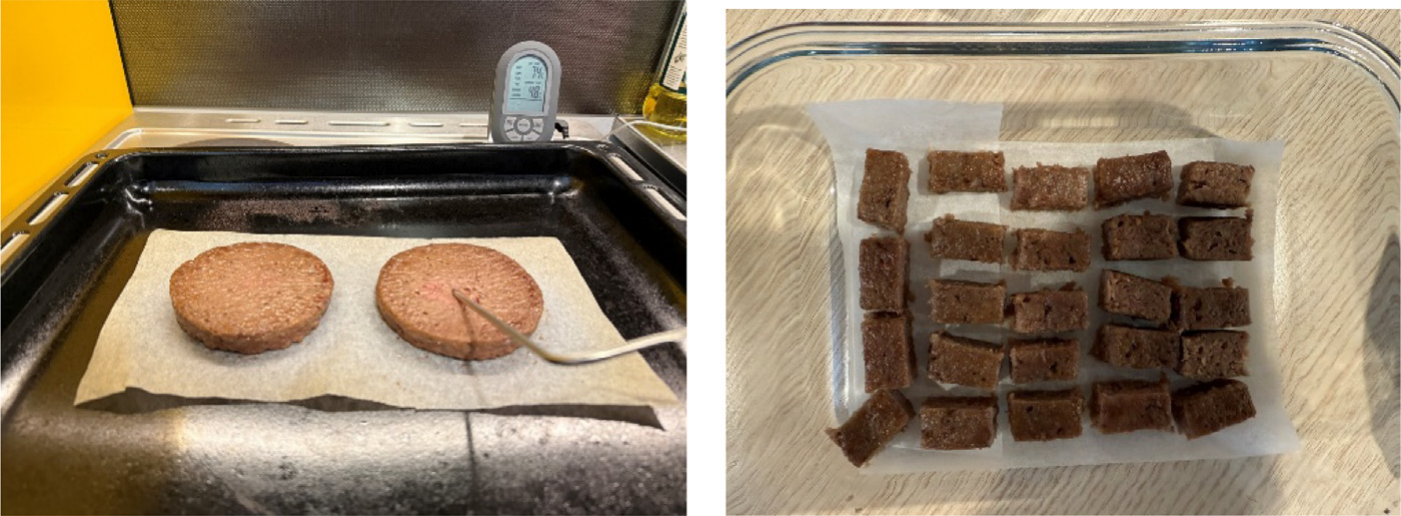
Cooked plant-based burger patties and samples cut into 2 g

This multimodal masticatory data collection experiment has been approved by The University of Auckland Human Participants Ethics Committee (Ethics Reference: UAHPEC26909). Before the experiment was carried out, we provided volunteers with detailed information about the data collection methods, the purposes of use, and the applications of the data, and successfully obtained their written consent. Three male participants (aged 29) were recruited based on the following inclusion criteria: good general health (self-reported), no dental or swallowing impairments, normal taste and smell perception, non-smoking status, non-vegetarian/vegan dietary habits, and willingness to consume both beef and plant-based burger patties. Fig. 9

### 4.3 Invivo chewing experiments

Each participant was first instructed to naturally chew and swallow three samples of each food type (beef and plant-based) using one side of the mouth. The chewing process was recorded to determine the number of chewing cycles before swallowing, i.e., the swallowing threshold for each participant. Subsequently, another three samples of each food type were provided, which participants chewed to their swallowing threshold but spat out just before swallowing.

### 4.4 Invivo post-chew analysis

The expectorated food boluses were collected, weighed, and imaged using (1) an iPhone 13 mini with 2800×2800 pixels, .JPG format under controlled lighting conditions, and (2) a Ricoh color scanner with 3496×4964 pixels, .JPEG format with consistent flatbed positioning. TPA was then performed to assess key textural parameters of the bolus at the point of near-swallowing. The acquired images and TPA data are available in the Invivo Data folder.

### 4.5 Biomimicking chewing robot

Invitro chewed food boluses were collected using the biomimicking chewing robot, ChewBot which has 3-DOF (degree-of-freedom) equipped with a soft robotic oral cavity [3]. The chewing robot simulates human mastication by replicating molar crushing and grinding motions, generating a series of molar chewing trajectories. The molar trajectories, from T1 to T13, provide increasing lateral displacement during occlusion, which result in greater shearing effect. T1 or the first trajectory is more of a vertical motion with more focus on biting and compression. T13 or the last trajectory is more of a lateral motion with more focus on grinding and shearing. The robotic oral cavity provides the function of containing the food and repositioning it back between occlusions by linear extension and contraction of pneumatic bellows. The saliva injected into the robot’s oral cavity is controlled by an automatic syringe to achieve a user-specified injecting rate.

### 4.6 ChewBots’ vision system

**Fig. 10:**
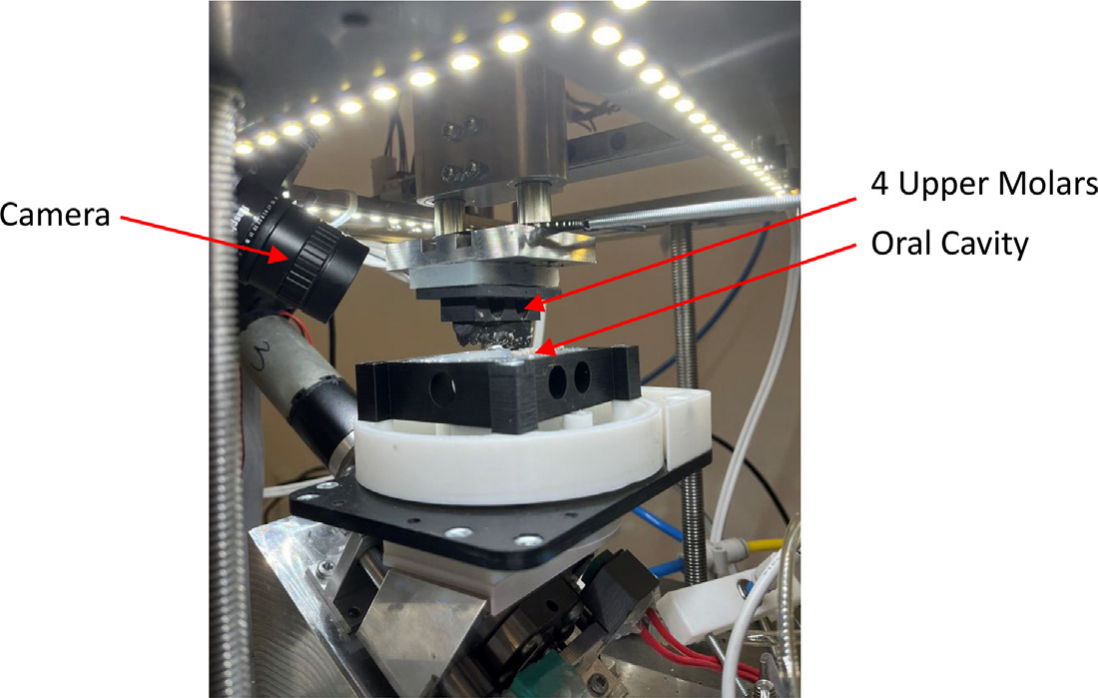
Camera setup.

The vision system uses a high-resolution Raspberry Pi HQ camera (2.1 MP) equipped with a 6 mm lens and an LED ring light to ensure consistent illumination as shown in Fig. 10. The camera is mounted around 10 cm away at a fixed oblique angle to the side of the robotic oral cavity, providing a clear view of the food bolus and molars throughout the chewing cycle. With a depth of field starting from 1 cm, the system captures real-time detailed images of the food bolus inside the robotic oral cavity. This setup enables the extraction of subtle textural and structural changes that occur as chewing progresses. The camera is synchronized with the robot’s chewing motion, capturing an image precisely when the upper jaw reaches full opening at each chewing cycle.

### 4.7 Invitro post-chew analysis

Three types of data were collected from these experiments and are available in Invitro Data folder.

1. An image of the chewed food bolus captured as soon as the robotic jaw opens up. Hence, for each experiment with 5, 10, 15, 20, 25, 30, 35, and 40, the same number of images were captured as well.
2. Force profiles (Newtons) recorded every 100 ms during each chewing cycle.
3. TPA metrics for chewed food boluses at each 5, 10, 15, 20, 25, 30, 35, and 40 chewing cycles.

## Limitations

• Small participant pool: The Invivo data were collected from only three male participants, limiting generalizability.
• The number of samples per chewing cycle and between food types may introduce bias during machine learning model training: The Invitro experiments used a limited range of chewing cycles (5 – 40), potentially missing intermediate breakdown stages and chewing cycles exceeding 40.
• Only two types of food: The chewing experiments can be extended to a range of food types in the future.
• The current imaging setup is the fixed side-angled camera position which limits the capture of certain features visible from a top-down or multi-angle perspective: Future versions could incorporate additional camera views or 3D reconstruction techniques.

## Ethics Statement

This is to confirm that, The University of Auckland Human Participants Ethics Committee (UAHPEC) approval was obtained for the Invivo chewing experiments. The reference number is UAHPEC26909. The dataset does not contain personally identifiable information, ensuring privacy and anonymity.

## CRediT Author Statement

**Isurie Akarawita:** Investigation, Methodology, Formal analysis, Writing - original daft. **Bangxiang Chen:** Methodology, Writing - review & editing, Supervision. **Jaspreet Singh Dhupia:** Investigation, Writing - review & editing, Supervision. **Martin Stommel:** Methodology, Writing - review & editing. **Weiliang Xu:** Methodology, Supervision, Project administration, Writing - review & editing.

## Data Availability

Mendeley DataChewNet: Dataset for Invivo and Invitro Beef and Plant-based Burger Patty Boluses (Original data).
